# Pathway incompatibility between NF-κB and RAS signaling constrains oncogenicity in B-cell leukemia

**DOI:** 10.1016/j.jbc.2026.113537

**Published:** 2026-09-08

**Authors:** Emma Stewart, Adriana Collins, Benjamin Kovacic, Mia Knupke, Grace M. Glasser, Hua Lou, Lai N. Chan

**Affiliations:** 1 Department of Heart, Blood and Kidney Research, Cleveland Clinic, Cleveland, Ohio, USA; 2 Department of Cancer Sciences, Cleveland Clinic, Cleveland, Ohio, USA; 3 Department of Genetics and Genome Sciences, Case Western Reserve University School of Medicine, Cleveland, Ohio, USA; 4 Department of Molecular Medicine, Cleveland Clinic Lerner College of Medicine, Case Western Reserve University School of Medicine, Cleveland, Ohio, USA

**Keywords:** cancer biology, signaling, oncogene, tumor suppression, leukemia

## Abstract

Oncogenic pathways do not always cooperate; in some contexts, their coactivation is antagonistic and suppresses tumorigenesis, a phenomenon we termed pathway incompatibility. However, the mechanisms underlying this antagonism and the role of receptor context in shaping these interactions remain unclear. During normal B-cell development, precursor B-cell receptor (pre-BCR) signaling supports survival and proliferation of early B-cell precursors before transition to expression of the mature B-cell receptor (BCR). B-cell acute lymphoblastic leukemia (B-ALL), the most common childhood cancer, is characterized by developmental arrest prior to BCR expression, and approximately 35% of cases harbor activating RAS–ERK mutations that mimic pre-BCR–dependent survival signaling. NF-κB plays context-dependent roles in B-cell malignancies, but whether it influences the compatibility between oncogenic RAS signaling and BCR expression remains poorly understood. Activation of canonical NF-κB–induced apoptotic depletion of RAS-driven B-ALL cells. Mechanistically, NF-κB suppressed pre-BCR–dependent survival signaling while promoting expression of BCR components. Consistent with this shift, oncogenic RAS signaling was poorly tolerated in BCR-positive cells unless BCR expression was disrupted. Pharmacologic activation of NF-κB reduced ERK signaling and selectively impaired viability of RAS-driven B-ALL cells, with enhanced effects in combination with ERK inhibition. Together, these findings show that canonical NF-κB signaling promotes BCR expression, which constrains oncogenic RAS activity, and establish pathway incompatibility as a mechanism through which receptor context can limit oncogenic potential.

Cancer progression is often viewed as the result of cooperating oncogenic signaling pathways (1, 2), whereas mutually exclusive pathway activation is typically interpreted as reflecting functional redundancy (3, 4). However, we previously identified an alternative scenario in B-cell acute lymphoblastic leukemia (B-ALL) termed “pathway incompatibility,” in which coactivated oncogenic pathways are antagonistic rather than synergistic (5). Activating mutations in STAT5 and RAS-ERK pathways are mutually exclusive in clinical B-ALL cases and exhibit reciprocal inhibition at the signaling level. Instead of cooperating to promote leukemogenesis, coactivation of these pathways suppresses it, revealing a tumor-suppressive mechanism based on antagonism between oncogenic signaling programs. This antagonism was associated with opposing transcriptional programs and distinct B-cell developmental cues (5), but the underlying mechanisms remain incompletely defined. Furthermore, its relevance beyond STAT5 and RAS signaling remains unknown.

B-ALL provides a tractable system to investigate these questions. B-ALL is one of the most common malignancies of childhood, with more than 5000 new cases diagnosed annually in the United States (6, 7, 8). The disease is characterized by disruption of normal B-cell differentiation, leading to accumulation of immature lymphoid precursors (9, 10). During normal B-cell development, early B-cell precursors express the precursor B-cell receptor (pre-BCR), composed of an immunoglobulin heavy chain paired with surrogate light chains (SLC; VpreB and λ5), which supports survival and proliferation. Replacement of surrogate with conventional light chains (cLCs) generates the mature B-cell receptor (BCR), enabling antigen recognition and immune function (11, 12). In B-ALL, leukemic cells are typically arrested prior to this transition, thereby evading differentiation signals while sustaining proliferative capacity (9, 10). B-ALL subtypes reflect distinct stages of B-cell differentiation and retain signaling dependencies that support leukemic survival and proliferation.

Activating mutations in the RAS–ERK pathway occur in approximately 35% of B-ALL cases (5, 13, 14, 15) and functionally mimic pre-BCR-dependent signaling, sustaining leukemic cell survival and proliferation in the absence of normal B-cell maturation (5, 16, 17). Our prior work showed that RAS–ERK activation induces expression of BCL6, a transcriptional repressor with oncogenic functions, thereby recapitulating key aspects of pre-BCR signaling to promote survival and proliferation (5). Consistent with this, deletion of the pre-BCR adapter *Blnk* suppresses RAS-driven leukemogenesis (5). Clinically, RAS pathway activation is associated with therapy resistance, relapse, and leukemic infiltration of the central nervous system (13, 14, 15), underscoring the need to identify vulnerabilities linked to this developmental state.

NF-κB plays context-dependent roles across B-cell malignancies, functioning as either an oncogenic driver or tumor suppressor depending on cellular context (18, 19, 20, 21, 22). In Philadelphia chromosome–positive (*Ph*^+^) B-ALL, driven by the BCR-ABL1 fusion kinase, NF-κB activity is essential for malignant transformation, as its inhibition impairs cell growth and enhances tyrosine kinase inhibitor sensitivity (20, 21). In this context, NF-κB has been shown to counteract STAT5-driven transcriptional programs (22), highlighting the complexity and context dependence of NF-κB signaling in lymphoid transformation. However, the basis for these divergent effects remains poorly understood. We therefore investigated whether interaction between oncogenic RAS and canonical NF-κB signaling in RAS-driven B-ALL represents a form of pathway incompatibility through which pre-BCR- and BCR-associated signaling states constrain oncogenic pathway output and leukemogenic potential.

## Results

### Canonical NF-κB activation suppresses growth of RAS-driven B-ALL cells

NF-κB signaling has context-dependent functions across B-cell malignancies and can promote either oncogenic or tumor-suppressive effects (18, 19, 20, 21, 22). NF-κB signaling operates through 2 mechanistically distinct pathways: canonical and noncanonical. Canonical signaling activates the IKK2 (IKBKB or IKKβ)/NEMO complex to induce p65:p50–dependent transcription, whereas the noncanonical pathway engages NIK and IKK1 (CHUK or IKKα) to generate p52:RelB complexes (23, 24, 25). These pathways can have distinct and, in some contexts, opposing biological effects (26).

To determine how NF-κB signaling influences the growth of RAS-pathway–mutated B-ALL cells, we selectively activated each pathway in WT mouse B-cell precursor cells transformed with NRAS^G12D^, a model previously used to study pathway incompatibility in B-ALL (5). Activating mutations in NRAS are among the most common RAS-pathway lesions (39.6%) in B-ALL (13).

Non-canonical NF-κB signaling was activated by overexpression of WT NIK, which increases p52:RelB abundance and DNA binding (27, 28). Canonical NF-κB signaling was activated using a constitutively active IKK2 mutant (IKBKB S177E/S181E; Ikk2ca) (29, 30). NIK overexpression promoted cell growth, whereas activation of canonical NF-κB through Ikk2ca resulted in depletion of cells in growth competition assays (Fig. 1*A*). This depletion was accompanied by a 2-fold increase in Annexin V–positive, SYTOX Blue–negative cells, indicative of phosphatidylserine externalization in the absence of membrane permeabilization and consistent with induction of early apoptosis (Fig. 1*B*).

**Figure 1:**
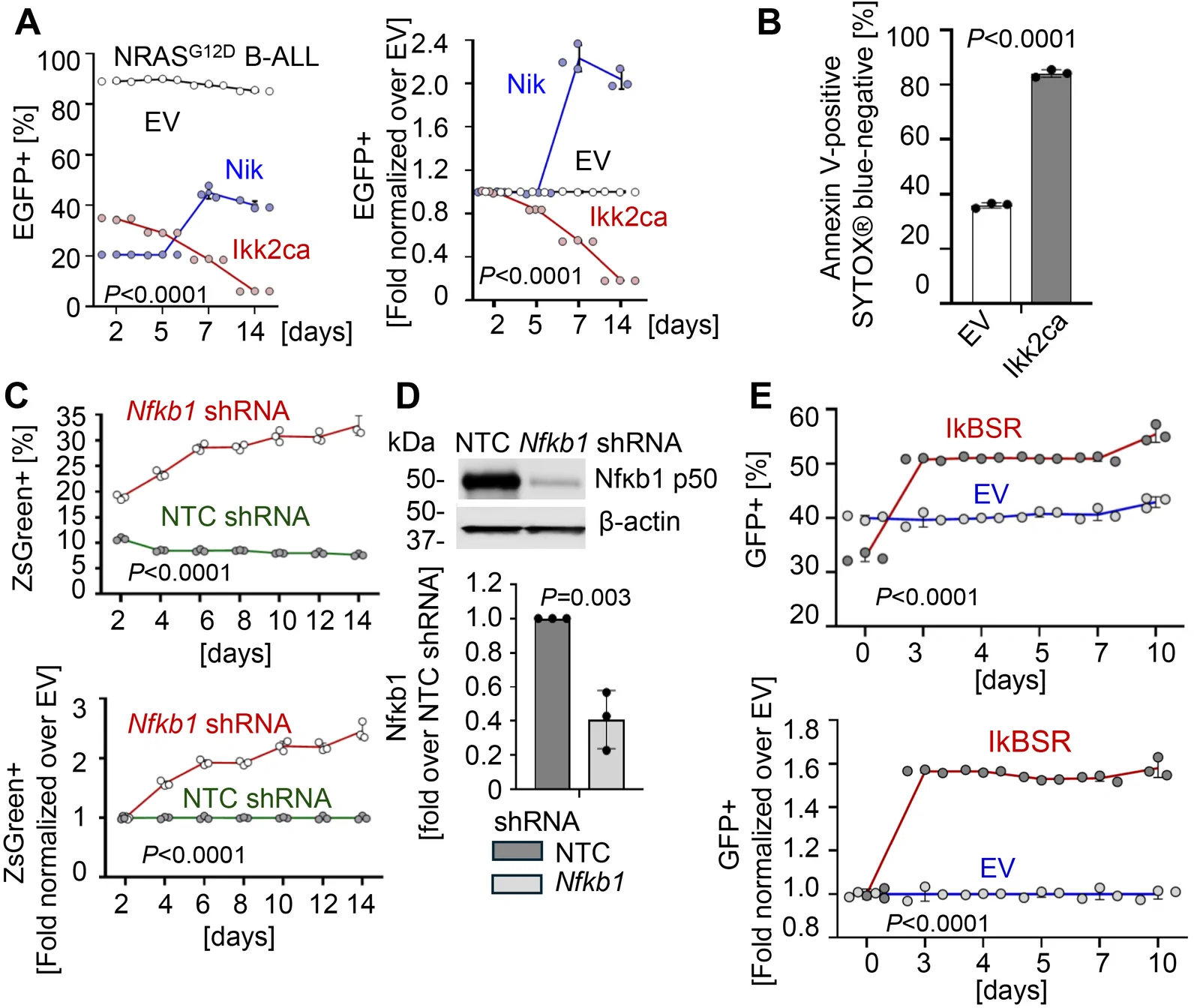
**Antagonism between ERK- and canonical NF-kB activation in RAS-pathway mutated B-ALL.***A*, enrichment or depletion of mouse NRAS^G12D^ B-ALL cells transduced with empty vector (EV) control, Ikk2ca-EGFP (constitutively active IKBKB mutant for canonical NF-κB activation), or NIK WT-EGFP (noncanonical NF-κB activation) as measured by flow cytometry at the indicated time points (n = 3 biological independent replicates; two-way ANOVA with Dunnett’s multiple comparisons test). *B*, five days following transduction with EV control or Ikk2ca, NRAS^G12D^ B-ALL cells were analyzed by flow cytometry for Annexin V-positive and SYTOX blue stain-negative populations, indicative of early apoptotic cells. Shown are data (mean ± SD) from three biological independent replicates and were analyzed by Welch’s *t* test. *C* and *D*, enrichment or depletion of mouse NRAS^G12D^ B-ALL cells transduced with ZsGreen-tagged nontargeting control (NTC) or *Nfkb1*-targeting shRNA, as measured by flow cytometry at the indicated time points (n = 3 biological independent replicates; *C*). Western blots of NRAS^G12D^ B-ALL cells expressing NTC shRNA or *Nfkb1*-targeting shRNA. Cells were sorted for Zs-Green-positive populations 2 days following transduction. Changes in Nfkb1 p50 were quantified after normalization to the loading control (β-actin). Data (mean ± SD, n = 3 independent biological replicates) are expressed as fold over NTC shRNA and analyzed by Welch’s *t* test (*D*). *E*, enrichment or depletion of NRAS^G12D^ B-ALL cells transduced with empty vector (EV) control or GFP-tagged IkBα super-repressor (IkBSR), n = 3 biological independent replicates, as measured by flow cytometry at the indicated time points. In panels *A*, *C*, and *E*, data are presented as the percentage of EGFP, ZsGreen, or GFP-positive cells and mean fold-change values normalized to the corresponding EV control at each time point. Data in panels *C* and *E* were analyzed by two-way ANOVA with Šidák’s multiple comparisons test. *p* values are indicated in the figures. ERK, extracellular signal-regulated kinase; NF-kB, nuclear factor kappa-light-chain-enhancer of activated B cells; B-ALL, B-cell acute lymphoblastic leukemia; IKBKB; inhibitor of nuclear factor kappa B kinase subunit beta.

Consistent with these findings, shRNA-mediated knockdown of *Nfkb1* conferred a competitive advantage (Fig. 1, *C* and *D*). Similarly, expression of a dominant-negative IκBα super-repressor, which blocks NF-κB nuclear translocation, promoted cell growth (Fig. 1*E*). Together, these findings indicate that canonical NF-κB activation suppresses growth of RAS-driven B-ALL cells, at least in part through induction of apoptosis.

### Canonical NF-κB and RAS–ERK signaling exhibit reciprocal antagonism

To investigate the relationship between canonical Nuclear factor kappa-light-chain-enhancer of activated B cells (NF-kB) and RAS-ERK signaling, we examined pathway activity in mouse B-cell precursor cells. NRAS^G12D^ expression in mouse B-cell precursor cells increased Erk1/2 phosphorylation while reducing phosphorylation of NF-κB p65 (Fig. 2*A*). Conversely, activation of canonical NF-κB through Ikk2ca enhanced p65 phosphorylation and decreased Erk1/2 phosphorylation (Fig. 2*B*). Together, these results demonstrate reciprocal antagonism between canonical NF-κB and RAS–ERK signaling pathways. Because these effects were specific to canonical NF-κB activation, subsequent analyses focus on this branch, hereafter referred to as NF-κB.

**Figure 2:**
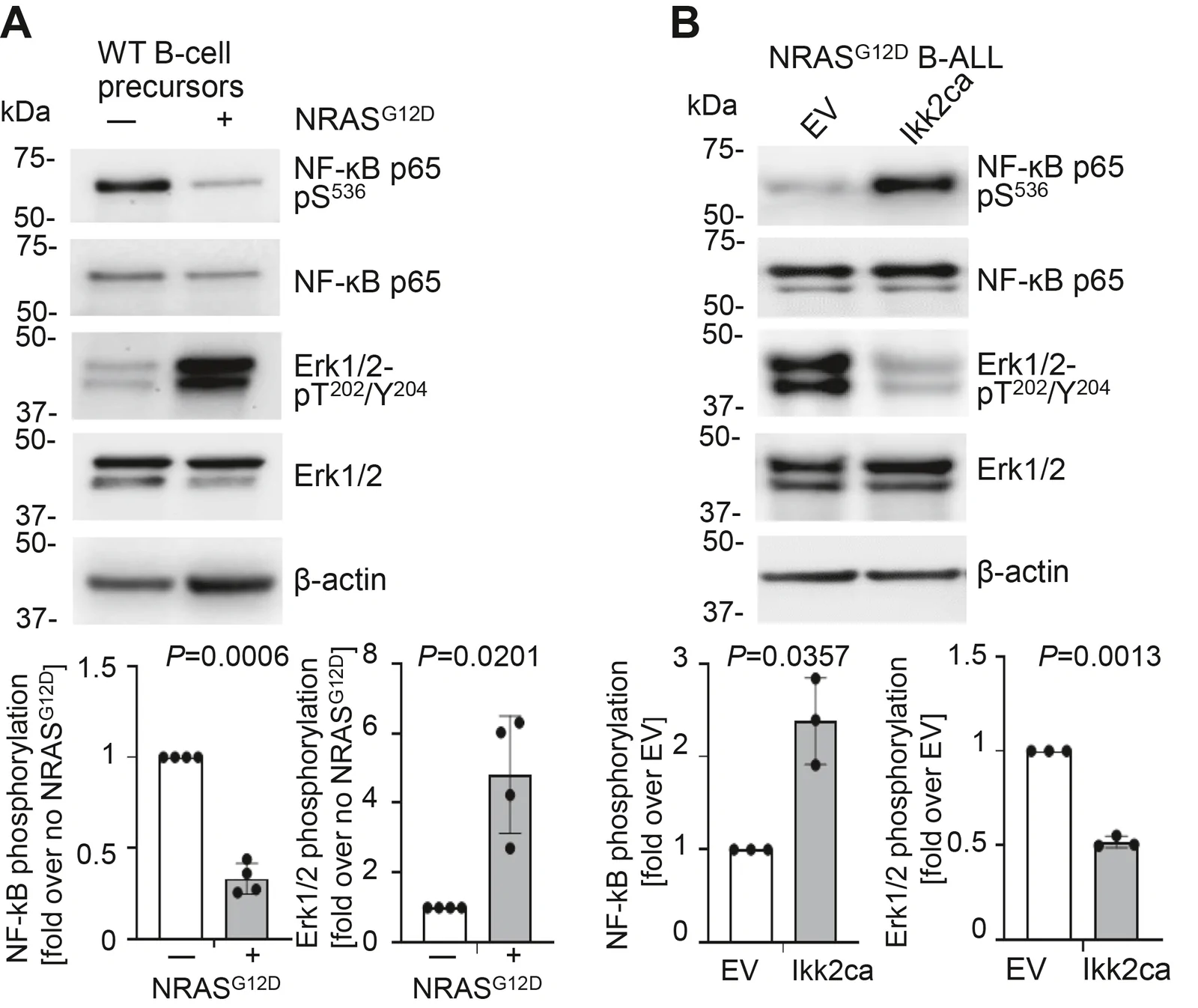
**Canonical NF-κB and oncogenic RAS display reciprocal signaling antagonism.***A*, Western blots of WT mouse B-cell precursor cells with or without NRAS^G12D^. *B*, Western blots of NRAS^G12D^ B-ALL cells expressing empty vector (EV) control or Ikk2ca (constitutively active IKBKB mutant for canonical NF-kB activation). NF-kB and Erk activation was determined 4 days after transductions. For Western blot quantification (*A* and *B*), phospho-Erk1/2 and total Erk1/2 signals were each normalized to the loading control (β-actin), and the normalized values were used to calculate the phospho/total Erk1/2 ratio. Similarly, phospho-NF-κB and total NF-κB signals were each normalized to β-actin, and the normalized values were used to calculate the phospho/total NF-κB ratio. Data (mean ± SD; n = 4 independent biological replicates in *A*, and n = 3 independent biological replicates in (*B*) are expressed as mean fold-change values relative to WT (*A*) or EV (*B*) and were analyzed using Welch's *t* test. *p* values are indicated in the figures. NF-kB, nuclear factor kappa-light-chain-enhancer of activated B cells; B-ALL, B-cell acute lymphoblastic leukemia; IKBKB; inhibitor of nuclear factor kappa B kinase subunit beta; ERK, extracellular signal-regulated kinase.

To determine whether this antagonism extends to an independent genetic model of elevated RAS-ERK signaling, we next compared WT B-cell precursor cells with *Nf1 23aIN/23aIN* cells, derived from a mutant mouse model in which altered alternative splicing pattern of *Nf1* exon 23a reduces neurofibromin Ras-GAP activity and selectively enhances RAS–ERK signaling without significantly affecting PI3K–AKT signaling (31). Consistent with prior studies, *Nf1 23aIN/23aIN* B-cell precursor cells showed elevated Erk1/2 phosphorylation relative to WT cells (Fig. 3, *A* and *B*). Notably, elevated Erk activity was accompanied by reduced phosphorylation of NF-κB p65 (Fig. 3, *A* and *B*), consistent with the reciprocal antagonism observed in NRAS^G12D^ cells (Fig. 2).

**Figure 3:**
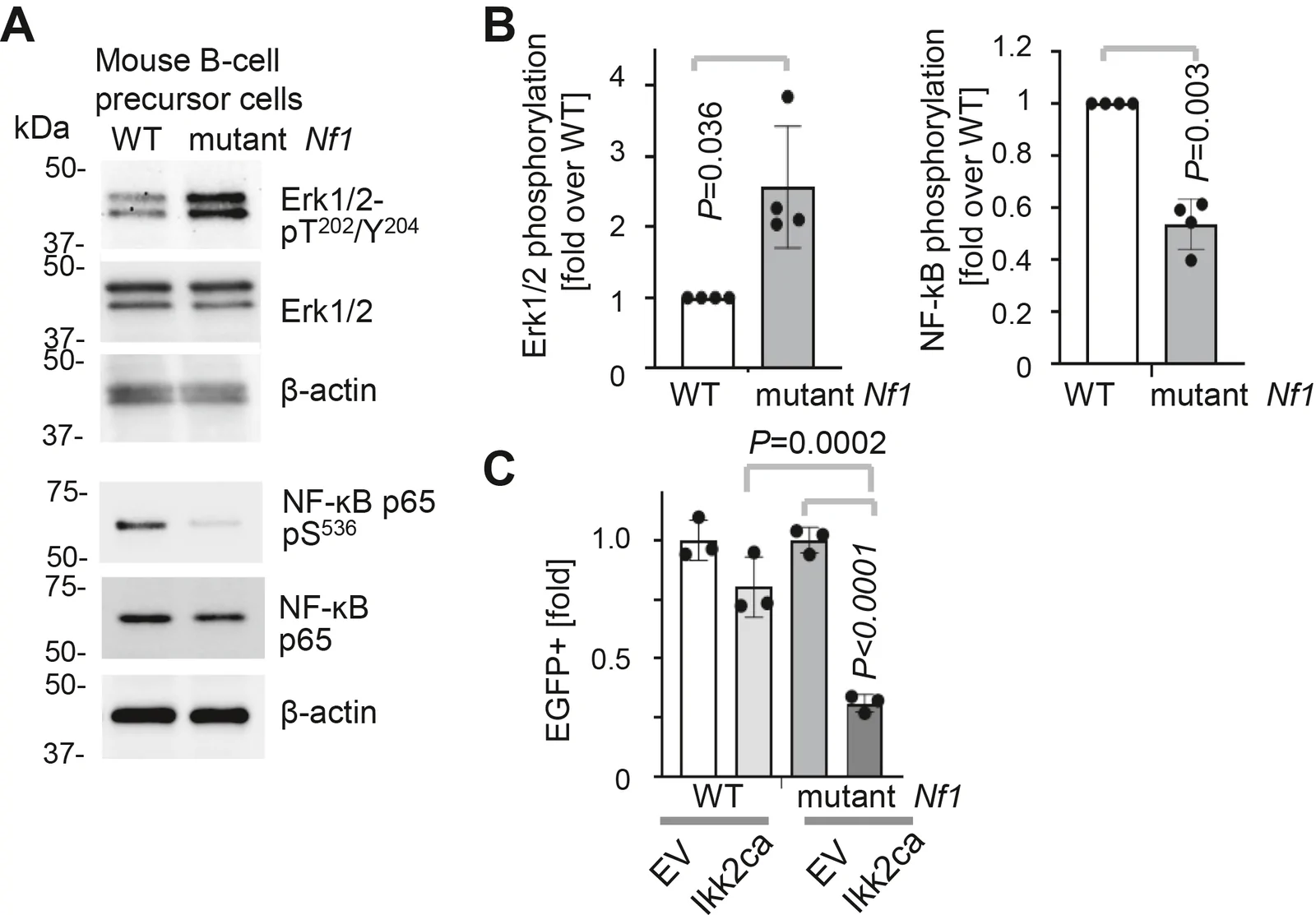
**Canonical NF-κB–mediated growth suppression depends on ERK signaling level.***A* and *B*, Western blots of mouse B-cell precursor cells showing low and high levels of Erk1/2 phosphorylation in WT and *Nf1 23aIN/23aIN* (*Nf1* mutant) B-cell precursor cells, respectively (*A*). For Western blot quantification, phospho-protein and total protein values were normalized to the loading control (β-actin), then the normalized values were used to determine the phospho/total ratios. Data (mean ± SD, n = 4 independent biological replicates) are expressed as mean fold-change values over WT and analyzed by Welch’s *t* test (*B*). *C*, relative enrichment or depletion of WT (low Erk activity) and *Nf1 23aIN/23aIN* (*Nf1* mutant; high Erk activity) mouse B-cell precursor cells following transduction with empty vector (EV) control or Ikk2 ca-EGFP (constitutively active IKBKB mutant for canonical NF-κB activation), measured by flow cytometry 5 days following transduction (n = 3 biological independent replicates). Data are presented as mean fold-change values relative to EV control and were analyzed by one-way ANOVA with Dunnett’s multiple comparisons test. *p* values are indicated in the figures. NF-kB, nuclear factor kappa-light-chain-enhancer of activated B cells; ERK, extracellular signal-regulated kinase; IKBKB; inhibitor of nuclear factor kappa B kinase subunit beta.

We next asked whether elevated Erk activity influences the response to canonical NF-kB activation. In growth competition assays, cells with higher Erk activity (*Nf1 23aIN/23aIN*) showed markedly greater depletion following Ikk2ca expression compared with cells displaying lower Erk activity (WT) (Fig. 3*C*). These findings indicate that the growth-suppressive effects of canonical NF-κB are enhanced in cells with elevated Erk activity.

### Canonical NF-κB attenuates pre-BCR–associated signaling

Our prior work showed that disruption of pre-BCR signaling suppresses the growth of RAS-driven B-ALL cells, indicating that pre-BCR activity is required for leukemogenesis (5). To determine how canonical NF-κB activation affects pre-BCR-associated signaling, we performed RNA-seq analysis following expression of Ikk2ca. Canonical NF-κB activation reduced expression of the SLC genes (*Vpreb1a* and *Vpreb3*) (Fig. 4*A*; GSE326596). Consistent with these findings, Ikk2ca reduced phosphorylation of the receptor-proximal kinase Syk (Tyr352), indicating attenuation of pre-BCR–associated signaling (Fig. 4, *B* and *C*).

**Figure 4:**
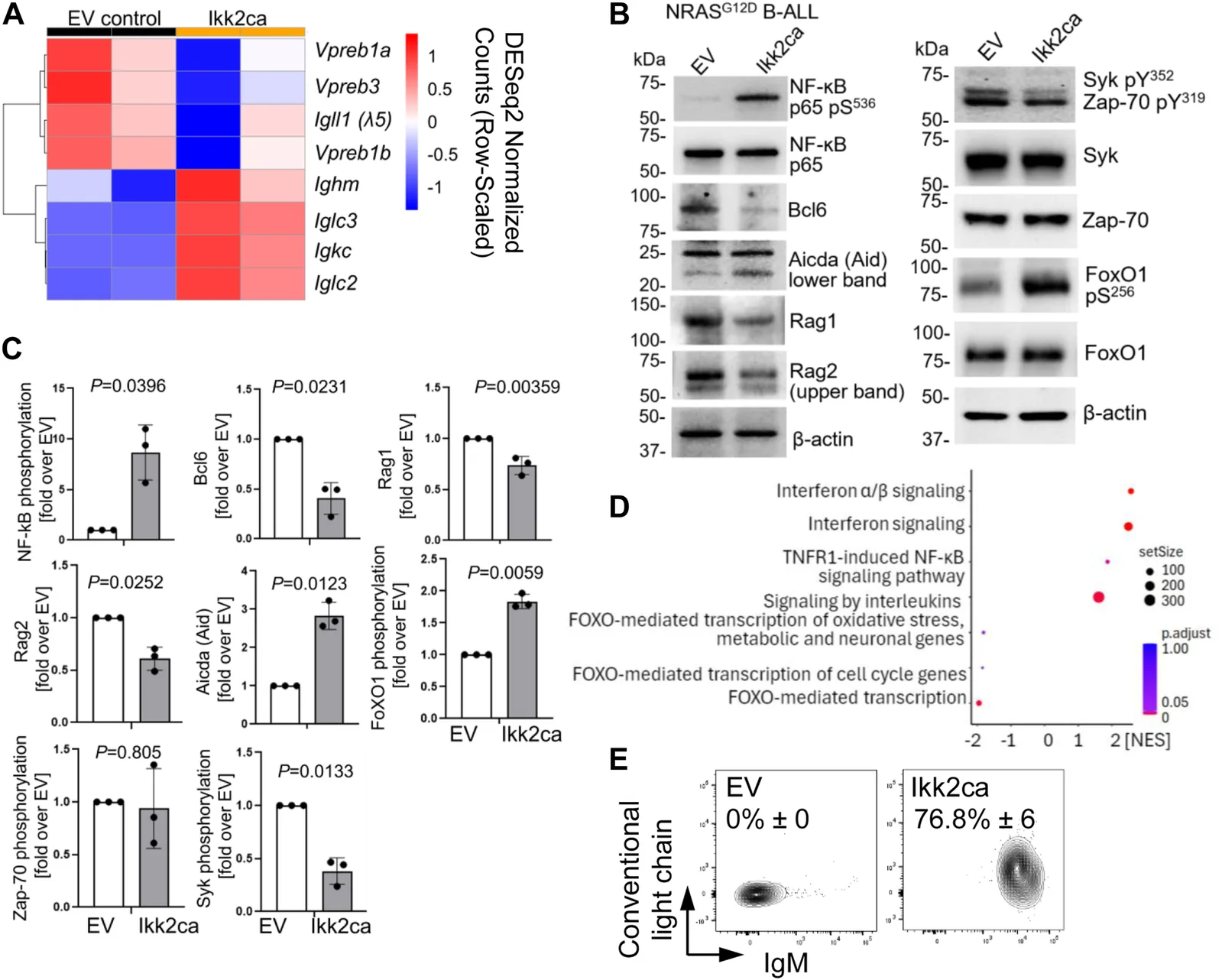
**Canonical NF-κB attenuates pre-BCR-associated signaling and promotes a shift toward a BCR-positive, more mature B-cell state.***A*, gene expression analysis of NRAS^G12D^ B-ALL cells expressing empty vector (EV) control or Ikk2ca (constitutively active IKBKB mutant for canonical NF-kB activation; 4 days following transduction). Heatmap of row-scaled DESeq2 normalized counts showcasing genes of interest *Ighm* (μ heavy chain), pre-BCR surrogate light chain components *(Vpreb1a, Vpreb3, Igll1*, and *Vpreb1b*), and BCR conventional light chain components (*Iglc3*, *Igkc*, and *Iglc2*). GEO accession number: GSE326596 (n = 2 independent biological replicates). *B* and *C*, Western blots of NRAS^G12D^ B-ALL cells expressing EV control or Ikk2ca (4 days following transduction; *B*). For quantification, phospho-protein and total protein values were normalized to β-actin, then the phospho/total ratios were determined using the normalized values. Changes in Bcl6, Aicda, Rag1, and Rag2 were quantified after normalization to β-actin. Data (mean ± SD; n = 3 independent biological replicates) are expressed as mean fold-change values relative to EV and were analyzed using Welch’s *t* test (*C*). *D*, pathway enrichment analysis of Foxo-mediated transcriptional programs and NF-κB pathways following canonical NF-κB activation with Ikk2ca in NRAS^G12D^ B-ALL cells. NES, normalized enrichment score (GEO accession number: GSE326596; same dataset as in panel *A*). *E*, Fflow cytometry analysis of surface expression of BCR components (conventional λ light chains and IgM) on mouse NRAS^G12D^ B-ALL cells upon overexpression of EV or Ikk2ca (4 days following transduction). Quantified results (percentage of double-positive cells) are shown as mean ± SD. Statistical significance was determined by Welch’s *t* test. NF-kB, nuclear factor kappa-light-chain-enhancer of activated B cells; pre-BCR, precursor B-cell receptor; BCR, B-cell receptor; IKBKB; inhibitor of nuclear factor kappa B kinase subunit beta; GEO, Gene Expression Omnibus.

NF-κB activation also increased phosphorylation of Foxo1 at S256 (Fig. 4, *B* and *C*), consistent with Foxo1 inactivation, and was accompanied by downregulation of FOXO target genes alongside upregulation of NF-κB–associated transcriptional programs (Fig. 4*D*). In line with these changes, Bcl6 expression was decreased (Fig. 4, *B* and *C*), consistent with reduced activity of signaling pathways that support pre-BCR–dependent survival. Together, these findings suggest that canonical NF-κB activation attenuates receptor-proximal signaling and key downstream effectors associated with the pre-BCR state.

### Canonical NF-κB promotes features associated with a BCR-positive state

Given that pre-BCR signaling defines an early developmental state, we next asked whether NF-κB activation promotes features of a more mature B-cell state. During normal B-cell development, SLCs are replaced by conventional κ or λ light chains, which pair with μ heavy chains to form the mature BCR, enabling B-cell differentiation (32, 33, 34, 35). In B-ALL, leukemic blasts are typically arrested prior to BCR expression, permitting continued proliferation and evasion of differentiation signals (9, 10).

Consistent with this developmental transition, our RNA-seq analysis (GSE326596) showed that canonical NF-κB activation increased expression of cLC genes (Fig. 4*A*). In contrast, inhibition of NF-κB signaling using IκBα super-repressor reduced expression of both pre-BCR and BCR components (Fig. S1), suggesting that NF-κB activity contributes to regulation of receptor component expression, while canonical activation is associated with a shift away from the pre-BCR-associated state. Flow cytometry analysis further demonstrated increased surface expression of cLCs and IgM (μ heavy chain) (Fig. 4*E*). Because coordinated expression and trafficking of both chains are required for receptor assembly, these findings are consistent with increased formation of surface BCR complexes.

Our RNA-seq analysis (GSE326596) further revealed that NF-κB activation altered expression of genes associated with B-cell developmental progression, including increased expression of surface markers and activation-associated genes linked to later stages of B-cell differentiation, such as *Ms4a1* (Cd20), *Cd40*, *Cr2* (Cd21), *Fcer2a* (Cd23), and activation-induced cytidine deaminase (*Aicda*), alongside reduced expression of *IL7r*, a receptor associated with early B-cell precursor survival, and the V(D)J recombination genes *Rag1* and *Rag2* (Fig. S2). These changes were validated at the protein level by flow cytometry (IL7r, Cd20, Cd40, Cd21, and Cd23; Fig. S3) and Western blotting (Aicda, Rag1, and Rag2; Fig. 4, *B* and *C*). Together, these findings indicate that canonical NF-κB activation disrupts signaling programs associated with the pre-BCR state while promoting expression of BCR components and selected features associated with later stages of B-cell differentiation.

### BCR expression constrains oncogenic RAS activity

Because canonical NF-κB activation increased expression of BCR components and promoted features associated with BCR expression, we next examined whether BCR expression influences tolerance of oncogenic RAS activity. Activating RAS-pathway mutations are enriched in malignancies arising from B-cell developmental stages lacking surface BCR expression, including B-ALL and multiple myeloma, but are less common in tumors derived from BCR-expressing B cells (36). Consistent with this pattern, oncogenic RAS efficiently transforms immature B-cell precursors in murine models (5) but is poorly tolerated in BCR-positive B cells (37, 38). These observations suggest that BCR expression may constrain oncogenic RAS activity.

We then tested whether oncogenic RAS is incompatible with the context of BCR expression using diffuse large B-cell lymphoma (DLBCL) cells that retain surface expression of BCR component conventional κ or λ light chains, key components of the BCR (Fig. 5*A*). Cells were transfected with empty vector control, or constructs expressing NRAS^G12D^ or KRAS^G12V^, the 2 most common activating RAS mutations in B-ALL (13). Growth competition assays were performed to determine whether sustained expression of oncogenic RAS is tolerated in this cellular context. Expression of either NRAS^G12D^ or KRAS^G12V^ led to depletion of cells in growth competition assays (Fig. 5, *B* and *C*), indicating that oncogenic RAS signaling is not well tolerated in this context.

**Figure 5:**
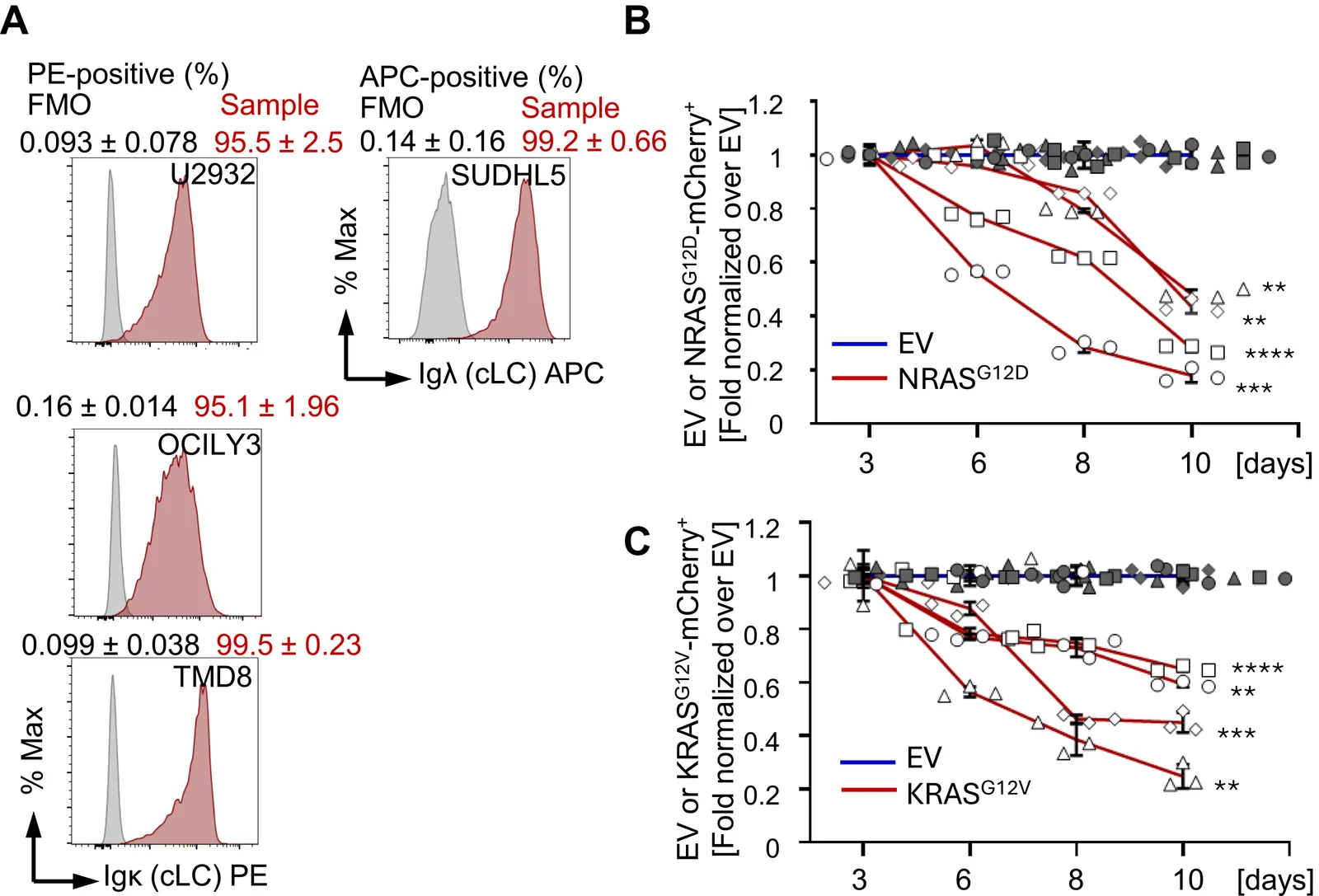
**Expression of oncogenic RAS depletes BCR-positive lymphoma cells.***A*, flow cytometry analysis of conventional κ or λ light chains (cLC) on human diffuse large B-cell lymphoma (DLBCL) cells. Surface expression of Ig κ or λ LC was shown on viable cells (SYTOX blue stain-negative). FMO: Fluorescence minus one (cells were stained with SYTOX *blue stain* but not PE-conjugated antibody against Ig κ LC or APC-conjugated antibody against Ig λ LC). Quantified results (mean ± SD) are from three independent experiments and were analyzed using Welch’s *t* test. OCILY3: *p* = 0.0001. SUDHL5: *p* < 0.0001. U2932: *p* = 0.0002. TMD8: *p* < 0.0001. *B* and *C*, cells were transfected with plasmid expressing mCherry-tagged empty vector (EV) control, or either NRAS^G12D^ (*B*) or KRAS^G12V^ (*C*) using electroporation. Enrichment or depletion of mCherry^+^ cells was monitored by flow cytometry at the indicated time points following transfection. Shown are average fold change values normalized to the corresponding control at each time point from three biological independent replicates. EV expressed: *blue line*, *gray symbol*. NRAS^G12D^ or KRAS^G12V^ expressed: *red line*, *white symbol*. U2932: *circle symbol*. OCILY3: *square symbol*. TMD8: *triangle symbol*. SUDHL5: *diamond symbol*. Statistical significance was determined by two-way ANOVA with Tukey’s multiple comparisons test. ∗∗*p* ≤ 0.01. ∗∗∗*p* ≤ 0.001. ∗∗∗∗*p* ≤ 0.0001. BCR, B-cell receptor.

To determine whether this effect was linked to BCR expression, we used CRISPR/Cas9 to disrupt the constant region of conventional κ light chains in U2932 and OCILY3 cells (Fig. 6*A*). In growth competition assays, NRAS^G12D^ or KRAS^G12V^ expression reduced the abundance of cells retaining conventional light-chain expression, whereas loss of cLCs significantly rescued this depletion (Fig. 6*B*). Together, these findings indicate that BCR expression constrains oncogenic RAS activity.

**Figure 6:**
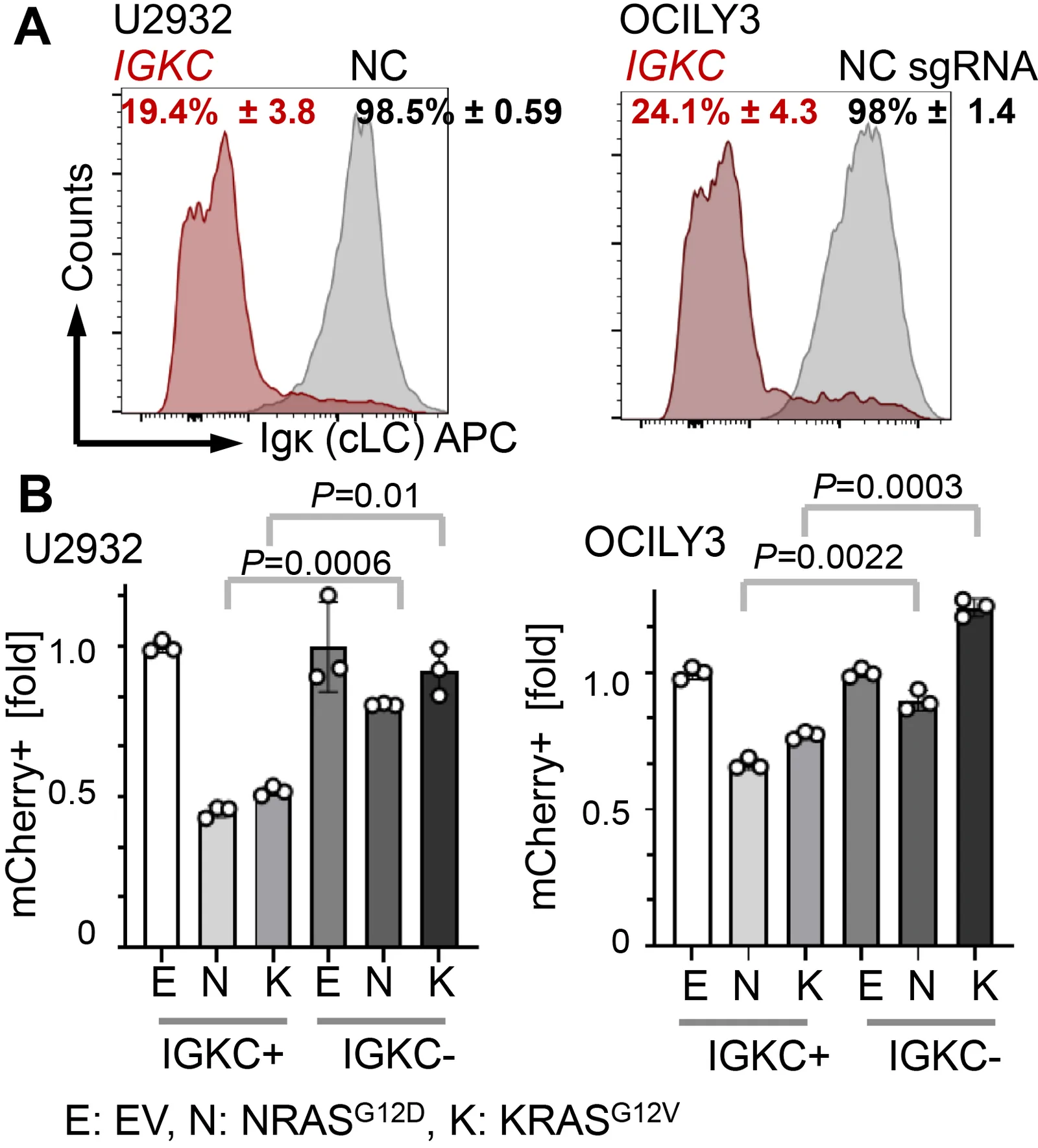
**BCR expression mediates intolerance to oncogenic RAS signaling.***A*, U2932 and OCILY3 cells were electroporated with Cas9 ribonucleoprotein (RNPs) complexes containing recombinant Cas9 and either nontargeting control control (NC) or conventional κ light chain (cLC; *IGKC*)-targeting guide RNAs. Flow cytometry was then performed to assess loss of surface expression of Ig κ LC to confirm *IGKC*-disruption 3 days following transfection. Quantified results (percentage of Ig κ cLC-positive cells) are shown as mean ± SD from four independent biological replicates. Statistical significance was determined by Welch’s *t* test. U2932 and OCILY3: *p* < 0.0001. *B*, two days following transfection, cells were sorted into IGKC-positive or IGKC-negative populations from NC or *IGKC* sgRNA-transfected cells, respectively. Two days following cell sorting, sorted cells were transfected with empty vector (EV) control, or constructs expressing NRAS^G12D^- or KRAS^G12V^-mCherry using electroporation. Enrichment or depletion of mCherry^+^ cells was measured by flow cytometry 5 days following electroporation (n = 3 biological independent replicates). Data are presented as mean fold-change values relative to the corresponding controls and were analyzed by Welch’s *t* test. *p* values are indicated in the figure. BCR, B-cell receptor; IGKC, immunoglobulin kappa constant.

### Pharmacologic activation of NF-κB recapitulates antagonism with RAS signaling in human B-ALL cells

We next examined whether the antagonistic interaction between NF-κB and RAS signaling observed in our system is reflected in cellular responses across human B-ALL models. Across a panel of human B-ALL cell lines (39, 40), sensitivity to canonical NF-κB activation correlated with baseline ERK pathway activity rather than RAS mutation status alone. The ERK-high 697 cells were preferentially depleted following IKK2ca expression, whereas Nalm-6, despite harboring an NRAS A146 T mutation, exhibited low baseline ERK activity and was not depleted. Similarly, RAS-WT cell lines with low ERK activity showed minimal response or relative enrichment (Fig. 7, *A* and *B*). To determine whether canonical NF-kB signaling was activated in IKK2ca-expressing 697 cells, we performed phospho-flow analysis. Increased phosphorylation of NF-kB p65 was detected on day four (Fig. 7*C*), when the earliest detectable depletion of IKK2ca-expressing cells became evident in growth competition assays (Fig. 7*B*). Thus, canonical NF-kB signaling was readily detectable under conditions in which growth inhibition was observed.

**Figure 7:**
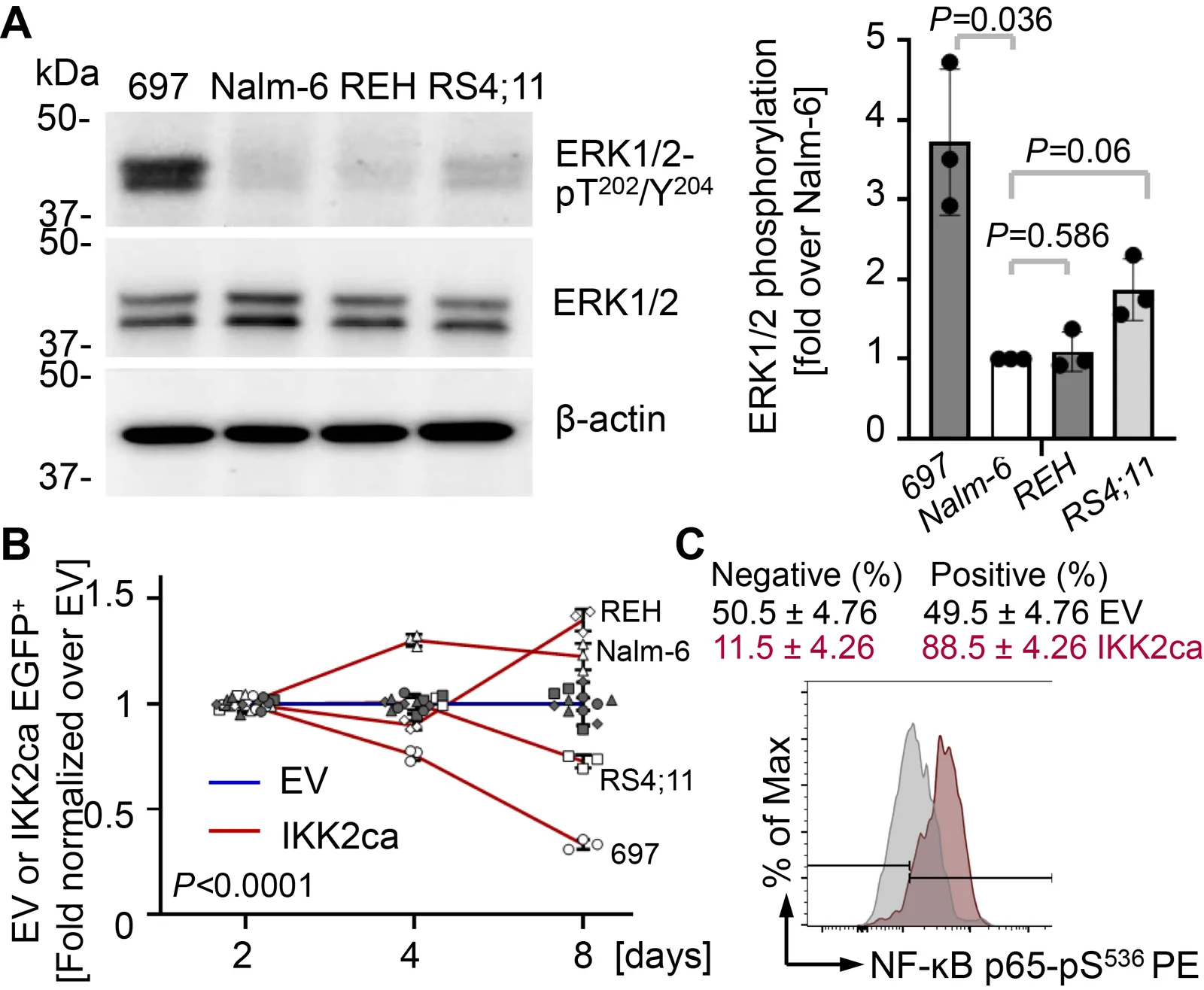
**ERK activity determines the outcome of NF-κB activation in human B-ALL cells.***A*, Western blots of human B-ALL cell lines. For Western blot quantification, phospho-ERK1/2 and total ERK1/2 values were normalized to the loading control (β-actin), then the normalized values were used to determine the phospho/total ratio. Data (mean ± SD, n = 3 independent experiments) are expressed as mean fold-change values over Nalm-6 and analyzed by Welch’s *t* test. Human B-ALL cell lines included 697 (NRAS^G12D^), Nalm-6 (NRAS^A146T^), RS4;11 (RAS WT), and REH (RAS WT). *B*, Human B-ALL cell lines were transfected with empty vector (EV; *blue line*, *gray symbol*) or IKK2ca-EGFP (constitutively active IKBKB mutant for canonical NF-κB activation; *red line*; *white symbol*) by electroporation (day 0). Enrichment or depletion of EGFP-positive cells was monitored by flow cytometry at the indicated time points following electroporation. Data are presented as mean fold-change values normalized to the corresponding EV control at each time point from three independent biological replicates. Statistical significance was determined by two-way ANOVA with Dunnett’s multiple comparisons test (*p* value indicated in the figure). A total of 697 cells: *circle symbol*; Nalm-6: *triangle symbol*; REH: *diamond symbol*; RS4;11: *square symbol*. *C*, flow cytometry analysis of phospho-NF-κB p65 (Ser536) in EV- or IKK2ca-expressing 697 cells on day four following electroporation. Cells were stained with a PE-conjugated phospho-NF-κB p65 (Ser536) antibody. Quantified results (mean ± SD) are from three independent biological replicates. *p* = 0.0005 (Welch’s *t* test). ERK, extracellular signal-regulated kinase; NF-kB, Nuclear factor kappa-light-chain-enhancer of activated B cells; B-ALL, B-cell acute lymphoblastic leukemia; IKBKB; inhibitor of nuclear factor kappa B kinase subunit beta.

To determine whether pharmacologic activation of NF-κB produces similar effects, we stimulated Toll-like receptor) signaling, which activates NF-κB through the MYD88 adapter pathway. Treatment with the Toll-like receptor 1/2 agonist CU-T12-9 (41) increased phosphorylation of NF-κB p65 while reducing ERK1/2 phosphorylation in RAS-pathway-mutated B-ALL cells (Fig. 8*A*). To determine whether this reduction reflected selective loss of ERK-high cells, we performed a phospho-flow time-course analysis following CU-T12-9 treatment. NF-κB activation preceded the decline in ERK phosphorylation and occurred before any substantial changes in cell viability were observed, supporting a direct suppressive effect of NF-κB signaling on ERK activity rather than selection against ERK-high cells (Fig. S4). Treatment with either CU-T12-9 or an ERK inhibitor alone reduced cell viability, and combined treatment produced a further decrease (Fig. 8*B*). These effects were selective for RAS-pathway-mutated contexts, as cells lacking RAS-pathway alterations showed minimal response (Fig. 8*C*).

**Figure 8:**
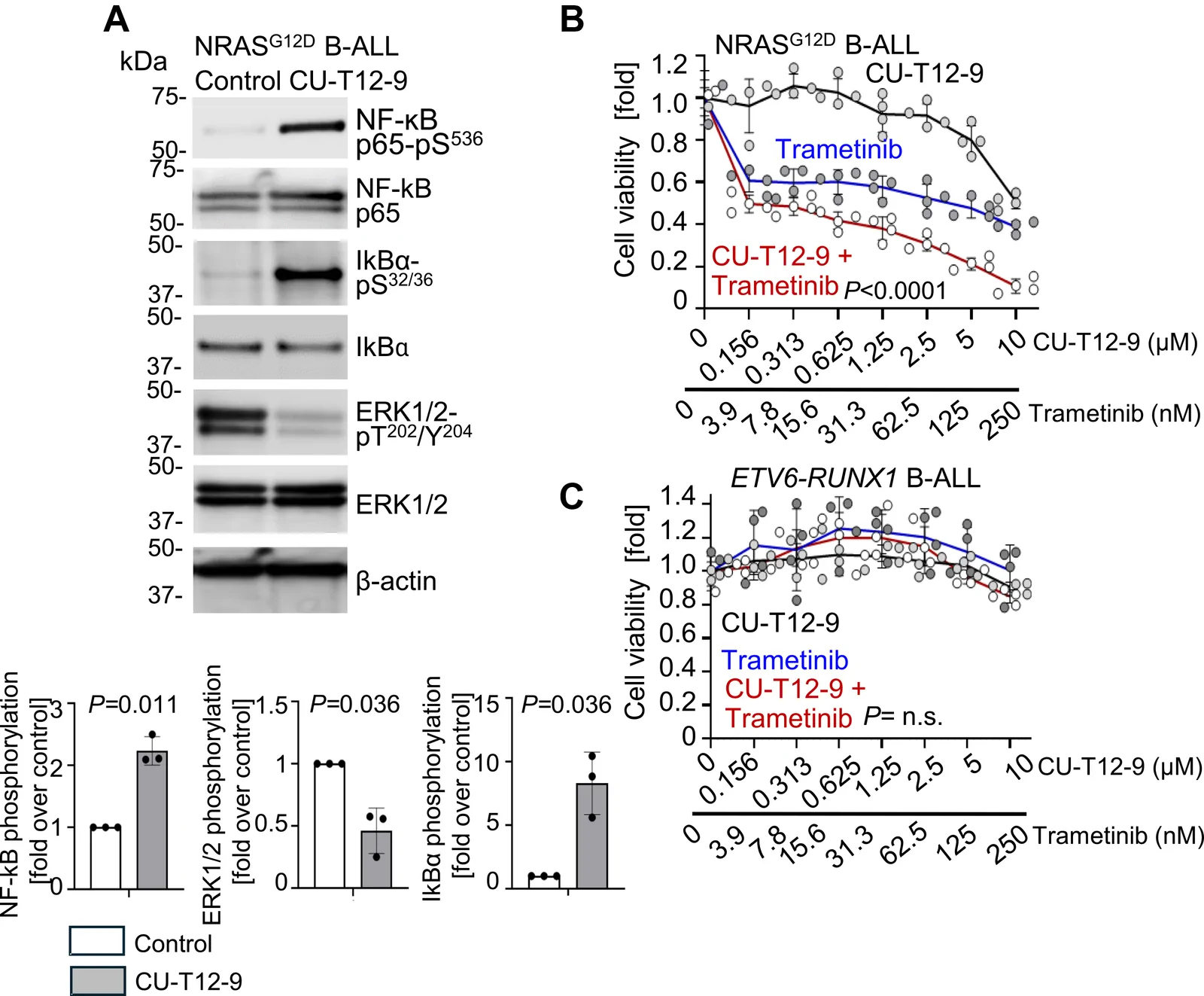
**Incompatibility between NF-κB- and RAS-pathways as a therapeutic target in B-ALL.***A*, Western blots of human NRAS^G12D^ B-ALL (697) cells following treatment with control or CU-T12-9 (10 µM; 48 h). For quantification, phospho-protein and total protein values were normalized to β-actin, next the phospho/total ratios were determined using the normalized values. Data (mean ± SD, n = 3 independent biological replicates) are expressed as fold over control and analyzed by Welch’s *t* test. *B* and *C*, human NRAS^G12D^ B-ALL (697) cells (*B*) and *ETV6-RUNX1* B-ALL (no RAS-pathway mutations, REH, *C*) cells were treated with vehicle control, CU-T12-9 alone (*black line*), trametinib (*blue line*) alone, a combination of both (*red line*) for 4 days. Cell viability was measured using CellTiter Glo assays. Shown are mean values of fold change relative to vehicle control (n = 4 independent biological replicates). CU-T12-9 (uM): 0, 0.156, 0.313, 0.625, 1.25, 2.5, 5, 10; Trametinib (nM): 0. 3.9, 7.8, 15.6, 31.3, 62.5, 125, 250. CU-T12-9-treated: *black line*, *light gray circle*. Trametinib-treated: *blue line*, *dark gray circle*. CU-T12-9 and trametinib-treated: *red line*, *white circle*. Statistical significance was determined by two-way ANOVA with Dunnett’s multiple comparisons test. *p* values are indicated in the figures. NF-kB, Nuclear factor kappa-light-chain-enhancer of activated B cells; B-ALL, B-cell acute lymphoblastic leukemia.

The observed antagonism between NF-κB and RAS–ERK signaling indicates that canonical NF-kB activation suppresses ERK signaling and impairs the fitness of RAS-driven B-ALL cells. Based on this model, we reasoned that combining activation of the incompatible NF-kB signaling program with direct pharmacologic inhibition of the primary oncogenic ERK pathway would provide complementary therapeutic pressures. Indeed, combined treatment with CU-T12-9 and an ERK inhibitor further reduced leukemia cell viability compared with either treatment alone, consistent with the concept that simultaneous inhibition of oncogenic ERK signaling and enforcement of an incompatible cellular program can more effectively constrain RAS-driven leukemia.

## Discussion

Oncogenic signaling pathways are commonly interpreted as cooperating to promote malignant transformation (1, 2), whereas mutually exclusive pathway activation observed across cancers is often attributed to functional redundancy (3, 4). The findings presented here support an alternative model in B-ALL in which specific signaling programs are incompatible and constrain leukemic fitness. Our data demonstrate that canonical NF-κB signaling antagonizes RAS–ERK activity and suppresses RAS-driven leukemogenesis, indicating that receptor-associated signaling context influences whether oncogenic pathways reinforce or oppose malignant growth.

Rather than functioning solely as a prosurvival pathway, canonical NF-κB signaling acted in this context as a suppressor of RAS-driven leukemic fitness. In contrast, activation of the noncanonical NF-κB pathway promoted cell expansion, highlighting distinct biological consequences of canonical and noncanonical NF-κB signaling in B-ALL. These findings further emphasize that the effects of NF-κB signaling are highly context dependent and cannot be generalized across B-cell malignancies.

Mechanistically, NF-κB-mediated suppression of RAS-driven leukemic fitness was associated with coordinated disruption of pre-BCR–associated signaling programs. Canonical NF-κB activation reduced expression of Bcl6, a critical effector previously shown to mediate pre-BCR–like signaling required for survival and proliferation of RAS-driven B-ALL cells (5). NF-κB activation was additionally associated with reduced Syk phosphorylation and suppression of FOXO-dependent transcriptional programs, supporting attenuation of signaling networks linked to the pre-BCR state. Concurrently, NF-κB activation increased expression of mature BCR components, consistent with a shift in receptor composition away from pre-BCR-associated signaling. Because oncogenic RAS functionally mimics pre-BCR signaling to sustain leukemic survival (5), these findings suggest that NF-κB antagonizes RAS-driven leukemogenesis, at least in part, by disrupting the receptor-associated signaling programs required for maintenance of the leukemic state.

Consistent with this model, activating RAS mutations were enriched in malignancies arising from developmental stages lacking surface BCR expression (36), and enforced expression of oncogenic RAS was poorly tolerated in BCR-positive lymphoma cells unless receptor expression was disrupted. These observations support the concept that compatibility between oncogenic signaling pathways is constrained by receptor context and help explain why oncogenic RAS preferentially supports transformation in cellular states lacking mature BCR expression.

The functional consequences of this incompatibility suggest potential translational relevance. Canonical NF-κB activation reduced ERK signaling and selectively impaired viability of RAS-pathway-activated B-ALL cells. Across both mouse and human models, sensitivity to canonical NF-κB activation correlated more closely with baseline ERK pathway activity than with RAS mutation status alone. Consistent with this, *Nf1*-mutant B-cell precursors, which exhibit elevated ERK signaling, showed enhanced sensitivity to canonical NF-κB activation, whereas the human B-ALL cell line NALM6, despite harboring an NRAS mutation, exhibited low baseline ERK activity and correspondingly limited sensitivity to IKK2ca. These findings suggest that ERK signaling output, rather than genotype alone, better predicts susceptibility to pathway incompatibility.

Importantly, the therapeutic rationale extends beyond reciprocal inhibition between NF-κB and ERK signaling. Within the pathway incompatibility framework, ERK inhibition directly targets the primary oncogenic driver, whereas NF-κB activation enforces a signaling and developmental state that is incompatible with maintenance of RAS-driven leukemic fitness. Consistent with this model, pharmacologic activation of NF-κB in combination with ERK inhibition further reduced leukemia cell viability, suggesting that simultaneously suppressing oncogenic signaling while promoting an incompatible signaling state may represent a complementary therapeutic strategy for RAS-driven B-ALL. More broadly, rather than relying solely on inhibition of the dominant oncogenic pathway, the pathway incompatibility framework predicts that therapeutic efficacy may be enhanced by simultaneously suppressing oncogenic signaling and activating a signaling program that is incompatible with the dominant oncogenic program.

Several questions remain. The molecular intermediates linking NF-κB activation to attenuation of ERK signaling and suppression of pre-BCR-associated programs remain incompletely defined and may involve transcriptional or chromatin-level regulation downstream of NF-κB. Whether canonical NF-κB directly regulates components of the pre-BCR signaling network or instead alters broader receptor-associated transcriptional programs will require further investigation. In addition, whether similar signaling constraints operate across genetically diverse leukemic contexts or other oncogenic pathway combinations remains to be determined. Whether similar constraints operate between other oncogenic signaling pathways also warrants investigation.

In summary, our findings demonstrate that canonical NF-κB signaling restricts RAS-driven leukemogenesis by suppressing pre-BCR-dependent survival programs and enforcing a BCR-associated signaling context incompatible with oncogenic RAS activity. These results provide mechanistic support for pathway incompatibility as a constraint on oncogenic signaling and identify receptor-associated signaling state as a key determinant of leukemogenic potential. More broadly, this work suggests that oncogenic signaling is not solely dictated by the presence of mutations but is critically shaped by the cellular signaling context in which these mutations operate. This framework provides a conceptual basis for identifying signaling programs that can be therapeutically exploited to constrain oncogenic function by enforcing incompatible cellular states.

## Experimental procedures

### Human leukemia and lymphoma cell lines

Human leukemia and lymphoma cells used in this study were listed in Table S1 and maintained in Roswell Park Memorial Institute medium (RPMI-1640; Invitrogen) with GlutaMAX containing 10%-20% FBS, 100 IU/ml penicillin and 100 μg/ml streptomycin. Cell cultures were kept at 37 °C in a humidified incubator with 5% CO2. Authenticity of the cell lines was validated using STR profiling.

### Extraction of bone marrow cells from mice

Bone marrow cells from 8 to 12 weeks old mice (Table S2) were harvested by flushing cavities of femur and tibia with chilled PBS followed by filtering through 40 μm strainer to yield a single-cell suspension. Filtered cells were further incubated with lysis buffer (RBC Lysis Buffer, BioLegend) to lyse erythrocytes. After washing with PBS, cells were subjected to further experiments. All mouse experiments were approved by the Institutional Animal Care and Use Committee of the Cleveland Clinic Research and Case Western Reserve University.

### Murine model of B-ALL

Bone marrow cells were harvested from 8 to 12 weeks old mice (Table S2) and cultured in Iscove’s modified Dulbecco’s medium (GIBCO) with GlutaMAX containing 20% FBS, 50 μM 2-mercaptoethanol, 100 IU/ml penicillin, 100 μg/ml streptomycin in the presence of 10 ng/ml recombinant mouse IL-7 (Peprotech). After 1 week of *in vitro* culture in the presence of IL-7, cells were confirmed to be nearly 100% Cd19 positive by flow cytometry. For the leukemia model, B cell precursor cells were retrovirally transduced with NRAS^G12D^-puromycin or NRAS^G12D^-K.orange followed by puromycin (1 μg/ml) selection or cell sorting for K.orange-positive populations, respectively. Cells were propagated only for short periods of time and usually not longer than 2 months to avoid acquisition of additional genetic lesions during long-term cell culture.

### Retroviral transduction

Constructs used for retroviral transduction are listed in Table S3. Retroviral supernatant was produced by transient cotransfection of Lenti-X 293T cells with retroviral constructs together with packaging plasmid pHIT60 (gag-pol) and envelop plasmid pHIT123 (5) (gifts from Dr Markus Müschen) using jetOPTIMUS. Triple-constructs transfected Lenti-X 293T cells were cultured on the poly-lysine-coated plate in high glucose Dulbecco’s Modified Eagle’s Medium (GIBCO) with GlutaMAX containing 10% fetal bovine serum, 100 μg/ml streptomycin, 100 IU/ml penicillin, 1 mM sodium pyruvate, and 0.1 mM non-essential amino acids for 16 h. To boost virus production, sodium butyrate (10 mM) was added to the culture for an additional 6 to 8 h and then washed out. The virus supernatant was collected 18 to 24 h after sodium butyrate induction and filtered through a 0.45 μm filter. For retroviral transduction, the non-tissue culture-treated plate was coated with 50 μg/ml Retronectin (Takara), and the virus-containing supernatants were loaded by centrifugation (2000*g*, 90 min at 32 °C). Two to 3 million cells per well were loaded on the plate by centrifugation at 600*g* for 30 min in the appropriate medium and cultured at 37 °C for 48 to 72 h. Cells transduced with constructs carrying an antibiotic resistance marker were selected with its respective antibiotic.

### Electroporation-based transfection

Transfection of two million cells with plasmids (5–10 μg) encoding genes of interest or empty vector controls (Table S3) was performed using the Neon NxT Transfection System (Invitrogen). Cells were resuspended in a Neon NxT buffer (100 μl) and subjected to electrical pulses to introduce plasmid DNA (Pulse: 1600V, width: 10 ms, number of pulses: three pulses). Following transfection, cells were returned to culture in the absence of penicillin and streptomycin for recovery. Cells were analyzed by flow cytometry 48 to 72 h posttransfection. Plasmids were purchased from VectorBuilder.

### shRNA knockdown of Nfkb1

Retroviral constructs carrying ZsGreen-tagged shRNAs targeting *Nfkb1 (*Table S4*)* or non-targeting control shRNA were purchased from Transomic Technologies. Retrovirus production and transductions were performed as described previously, followed by cell sorting to select cells expressing ZsGreen-tagged shRNA. Knockdown efficiency was verified by Western blot.

### CRISPR-mediated gene deletion

For non-viral gene deletion of conventional kappa light chains (Ig kappa LC, *IGKC*) in human cells, Alt-R CRISPR-Cas9 guide RNAs (Table S4) and non-targeting control guide RNAs (Cat # 1072544, 1072545, 1072546) were purchased from IDT. Chemically synthesized crRNAs (100 μmol/L) and tracrRNAs (100 μmol/L) were annealed by incubation at 95 C° for 5 min. Recombinantly produced Cas9 (40 μmol/L) were then added to RNA mixture to produce RNA ribonucleoprotein complexes. Electroporation (Pulse: 1600V, width: 10 ms, number of pulses: three pulses) was performed by using the Neon NxT Transfection system (Invitrogen). Gene deletion efficiency was verified by flow cytometry.

### Western blotting

Cells were lysed in CelLytic buffer (Sigma-Aldrich) supplemented with 1% protease inhibitor cocktail (Roche Diagnostics), 1% phosphatase inhibitor cocktail (EMD Millipore) and 1 mM PMSF on ice. A total of 15 to 20 μg of cell lysates per sample were separated on mini or midi precast gels (Bio-Rad) and transferred on nitrocellulose membranes using the Trans-Blot Turbo System (Bio-Rad). After blocking, membranes were probed overnight at 4 °C with the appropriate primary antibodies (Table S5) at the dilutions recommended by the manufacturers. The specificity of the antibodies was authenticated by Western blot analyses (including knockdown or KO samples when possible) and appropriate controls were included in every experiment. Next, membranes were incubated with alkaline phosphatase conjugated secondary antibodies (Invitrogen) and chemiluminescent substrate (Invitrogen) and were further detected by ChemiDoc^TM^ MP Imaging System (Bio-Rad). Densitometric analysis of Western blots was performed using Bio-Rad Image Lab Analysis Software. Briefly, .scn files were opened, lanes and bands were defined, background signal was subtracted, and band volume/intensity measurements were obtained. For Western blot quantification, phospho-protein and total protein values were normalized to the loading control (β-actin), then the normalized values were used to determine the phospho/total ratios. Changes in protein of interest, including Nfkb1, Bcl6, Aicda, Rag1, and Rag2, were quantified after normalization to the loading control (β-actin).

### Flow cytometry

For cell surface staining, approximately one million cells per sample were resuspended in PBS blocked using Fc blocker for 15 min on at 4 °C, followed by staining with the appropriate dilution of the antibodies, as recommended by the manufacturers, for 30 min at 4 °C. Cells were washed and resuspended in PBS with SYTOX Blue stain (1 μM) as a dead cell marker. Acquisition was performed on a LSRFortessa flow cytometer (BD Biosciences). The antibodies used for flow cytometry are listed in Table S5. For growth competition assays, SYTOX Blue-positive cells (dead cells) were excluded, and the proportions of viable EGFP^+^, GFP^+^, Zs-Green^+^ K.orange^+^, and mCherry^+^ cells were monitored. For Annexin V staining (detection of apoptosis), Annexin V binding buffer (Thermo Fisher Scientific) was used instead of PBS and SYTOX Blue stain (Thermo Fisher Scientific) was used as a dead cell marker. PE labeled-Annexin V was purchased from Thermo Fisher Scientific. For phospho-flow cytometry, cells were treated as indicated in the figure legends and fixed by adding an equal volume of pre-warmed Fixation Buffer (Biolegend), followed by incubation at 37 °C for 15 min. Cells were then washed, permeabilized with pre-chilled True-Phos Perm Buffer (Biolegend), and incubated at −20 °C for at least 60 min. After 2 additional washes, cells were resuspended in Cell Staining Buffer (Biolegend) and stained with fluorophore-conjugated phospho-specific antibody (Table S5) for 30 min at RT in the dark. Following final washes, cells were resuspended in Cell Staining Buffer and analyzed by flow cytometry. Fluorescence-based cell sorting was performed on FACSAria Fusion cell sorter (BD Biosciences). FACS data were analyzed with FlowJo software (FlowJo, LLC). Biexponential scaling was applied to the data.

### Cell viability assays

Forty thousand B-ALL cells were seeded in a volume of 80 μl in complete growth medium on 96-well plates (BD Biosciences). Compounds were added at the indicated concentration in a total volume of 100 μl. After culturing for 4 days, CellTiter-Glo 2.0 (Promega) assays were performed according to the manufacturer’s instructions. Medium without cells was used as blank. Relative viability was calculated using baseline values of cells treated with vehicle control as a reference.

### Statistical analyses

Data are presented as mean ± SD from 3 or more independent biological replicates. Statistical significance was determined using Welch’s *t* test, one-way ANOVA or two-way ANOVA followed by multiple comparisons as indicated in the figure legends, with *p* < 0.05 considered significant. Analyses were performed using Prism 11.0.2 (92) software (GraphPad).

### Bulk RNA-seq

Total RNA was extracted using NucleoSpin RNA Plus kit (Macherey-Nagel) according to manufacturer’s instructions. Libraries were constructed using the Illumina Stranded mRNA Prep kit. Samples were sequenced on the NovaSeq 6000 system. Raw reads in the FASTQ format were aligned to the latest available reference genome using STAR aligner 2.7.11a (42). RSeqQC was used to perform quality control and identify quality of RNA alignment from the bam files (43). The featureCounts read summarization program (44) was used to count mapped reads for each gene in the annotation file (Mouse GENECODE: Release M38 (GRCm39)). Count files were then merged by an in-house R script and differential gene expression analysis was performed using DESeq2 (45). DESeq2 normalizes the count data to account for differences in sequencing depth and composition. This is done using the median of ratios method, which involves dividing each gene's raw count by a size factor that accounts for library size and composition. DESeq2 models the variation in gene expression using a negative binomial distribution. The dispersion estimates are used to model the relationship between mean expression and variance, which is critical for accurate detection of differentially expressed genes (DEGs). DESeq2 uses a Wald test to assess the significance of differences in gene expression between groups. The test is based on a generalized linear model that includes terms for the mean expression and dispersion estimates. To account for the possibility of false positives due to multiple hypothesis testing, DESeq2 adjusts the *p*-values using the Benjamini-Hochberg procedure, which controls the false discovery rate. The final output of DESeq2 is a list of DEGs, along with their log2-fold changes and adjusted *p*-values.

### For Gene Set Enrichment Analysis (GSEA) dotplots

GSEA is a computational method that determines whether *a priori* defined set of genes shows statistically significant, concordant differences between 2 biological states. This list of DEGs was used to perform GSEA using R package clusterProfiler (46). GSEA analysis was implemented on Reactome genesets (47) downloaded from GSEA-MSigDB. The dotplot function from the enrichplot R package was used to visualize the pathway profiles and enrichment results for all reactome pathways (46).

## Data availability

All data are contained within the article. The RNA-seq dataset generated during this study are available in the Gene Expression Omnibus under accession number GSE326596. To review GEO accession GSE326596.

## Supporting information

This article contains supporting information (31, 48).

## Conflict of interest

The authors declare that they have no conflicts of interest with the contents of this article.
