# Supplementary material for: Pathway incompatibility between NF-κB and RAS signaling constrains oncogenicity in B-cell leukemia
Source: J Biol Chem. 2026 Sep 8;302(10):113537. doi: 10.1016/j.jbc.2026.113537 (PMC13671027; doi:10.1016/j.jbc.2026.113537)
Supplement: Supplementary file 1 [file mmc1.pdf]

Supporting Information

Pathway incompatibility between NF-κB and RAS signaling constrains oncogenicity in B-cell leukemia

Emma Stewart[1], Adriana Collins[1], Benjamin Kovacic[2], Mia Knupke[1], Grace M. Glasser[3], Hua Lou[3], Lai N. Chan[1,4]

[1]*Department of Heart, Blood and Kidney Research, Cleveland Clinic, Cleveland, OH 44195*

[2]*Department of Cancer Sciences, Cleveland Clinic, Cleveland, OH 44195*

[3]*Department of Genetics and Genome Sciences, Case Western Reserve University School of Medicine, Cleveland, OH 44195*

[4]*Department of Molecular Medicine, Cleveland Clinic Lerner College of Medicine, Case Western Reserve University School of Medicine, Cleveland, OH 44195*

Table of Content

Figure S1. NF-κB inhibition via IκBα-SR reduces expression of pre-BCR and BCR components.

Figure S2: NF-κB activation promotes expression of markers associated with a more mature B-cell state.

Figure S3: NF-κB activation promotes surface expression of markers associated with a more mature B-cell state.

Figure S4: NF-κB activation precedes ERK activity suppression following treatment with CU-T12-9

Table S1: Overview of leukemia and lymphoma cell lines used in this study.

Table S2: Overview of genetic mouse models used in this study.

Table S3: Mammalian gene expression vectors – regular plasmids and retrovirus constructs.

Table S4: Oligonucleotide sequences.

Table S5: Antibodies used in this study.

For correspondence:
Lai N. Chan, PhD
E-mail: chanl3@ccf.org
Cleveland Clinic Research
9500 Euclid Avenue, Cleveland, OH 44195

Running title: NF-κB constrains RAS-driven leukemia
Keywords: Cancer biology, signaling, oncogene, tumor suppression, leukemia

Figure S1. *NF-κB inhibition via IκBα-SR reduces expression of pre-BCR and BCR components*

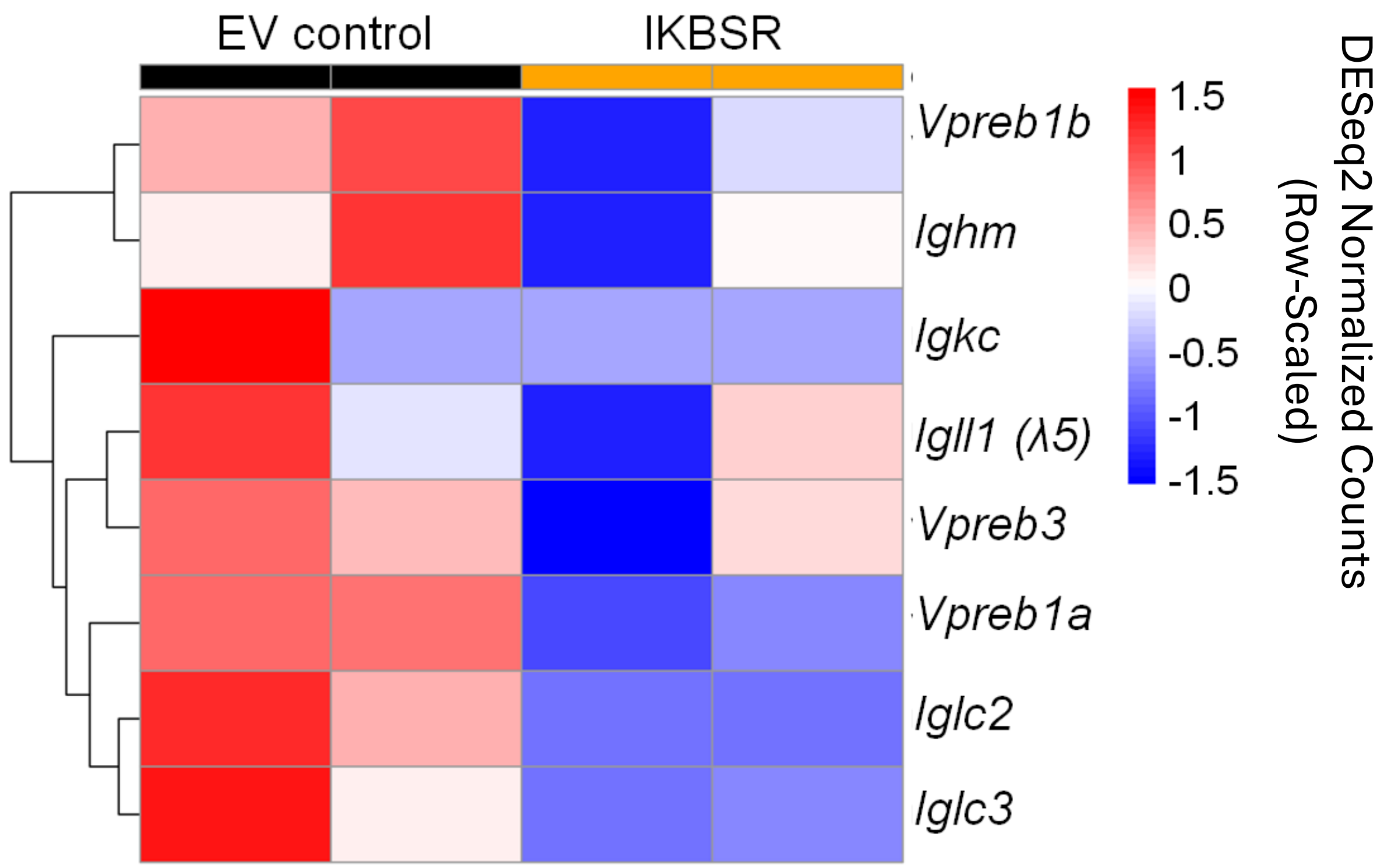

Gene expression analysis of NRAS^{G12D} B-ALL cells expressing empty vector (EV) control or IkBα-SR (NF-kB inhibition; 4 days following transduction). Heatmap of row-scaled DESeq2 normalized counts of *Ighm* (μ heavy chain), pre-BCR surrogate light chain components *(Vpreb1a, Vpreb3, Igll1* and *Vpreb1b*) and BCR conventional light chain components (*Iglc3*, *Igkc*, and *Iglc2*). GEO accession number: GSE326596 (n =2 independent biological replicates).

Figure S2. *NF-κB activation promotes expression of markers associated with a more mature B-cell state*

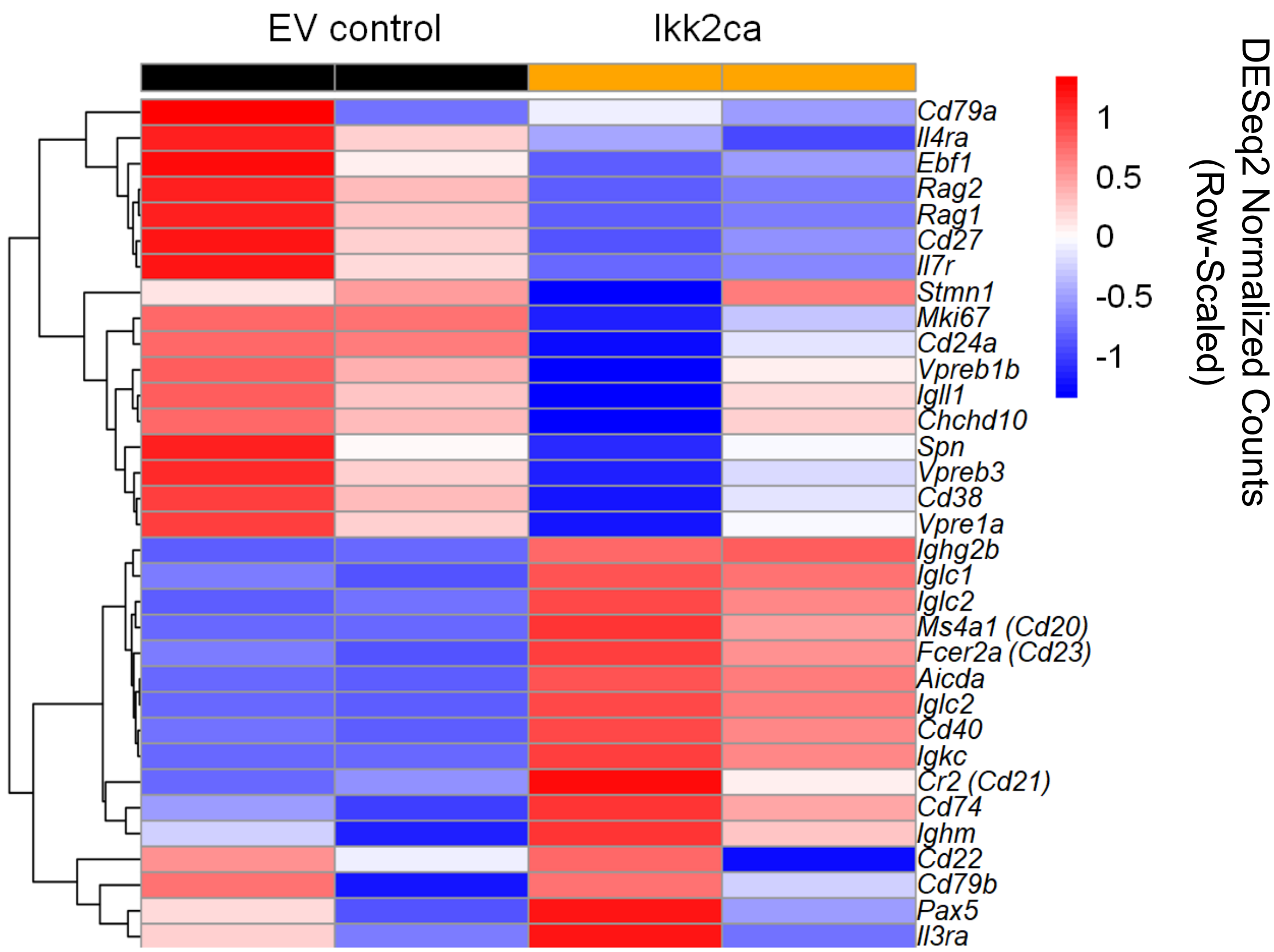

Gene expression analysis of NRAS^{G12D} B-ALL cells expressing empty vector (EV) control or Ikk2ca (constitutively active IKBKB mutant for canonical NF-kB activation; 4 days following transduction). Heatmap of row-scaled DESeq2 normalized counts of early and mature B-cell markers. GEO accession number: GSE326596 (n =2 independent biological replicates).

Figure S3. *NF-κB activation promotes surface expression of markers associated with a more mature B-cell state*

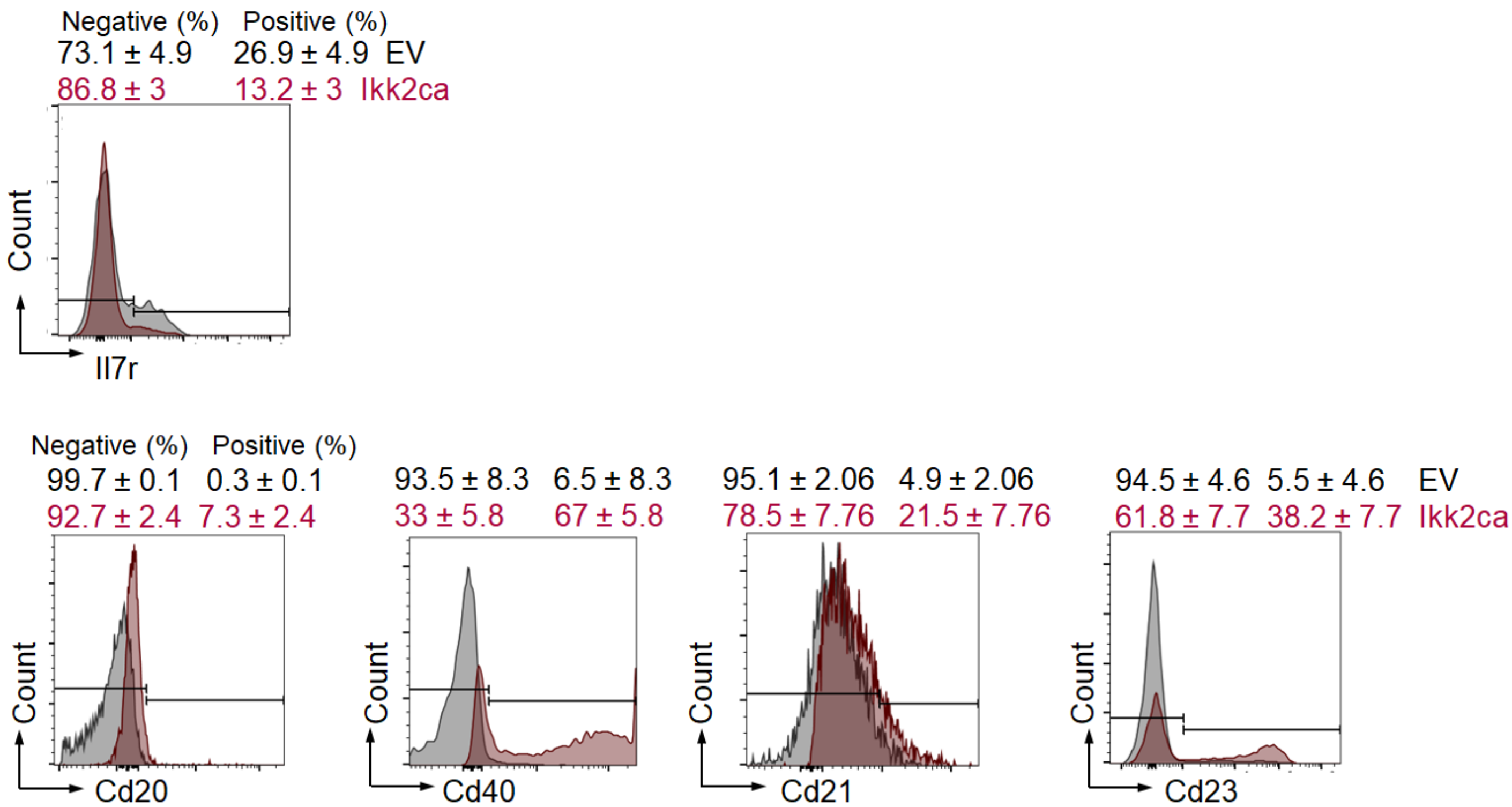

Flow cytometry analysis of surface expression of early as well as mature B-cell markers on mouse NRAS^{G12D} B-ALL cells upon overexpression of EV or Ikk2ca (4 days following transduction). Shown are representative results from 3 independent biological replicates. Statistical significance was determined by Welch's t-test. Il7r: $P = 0.0206$. Cd20: $P = 0.0372$. Cd40: $P = 0.0009$. Cd21: $P = 0.0096$. Cd23: $P = 0.0064$.

Figure S4. *NF-κB activation precedes ERK activity suppression following treatment with CU-T12-9*

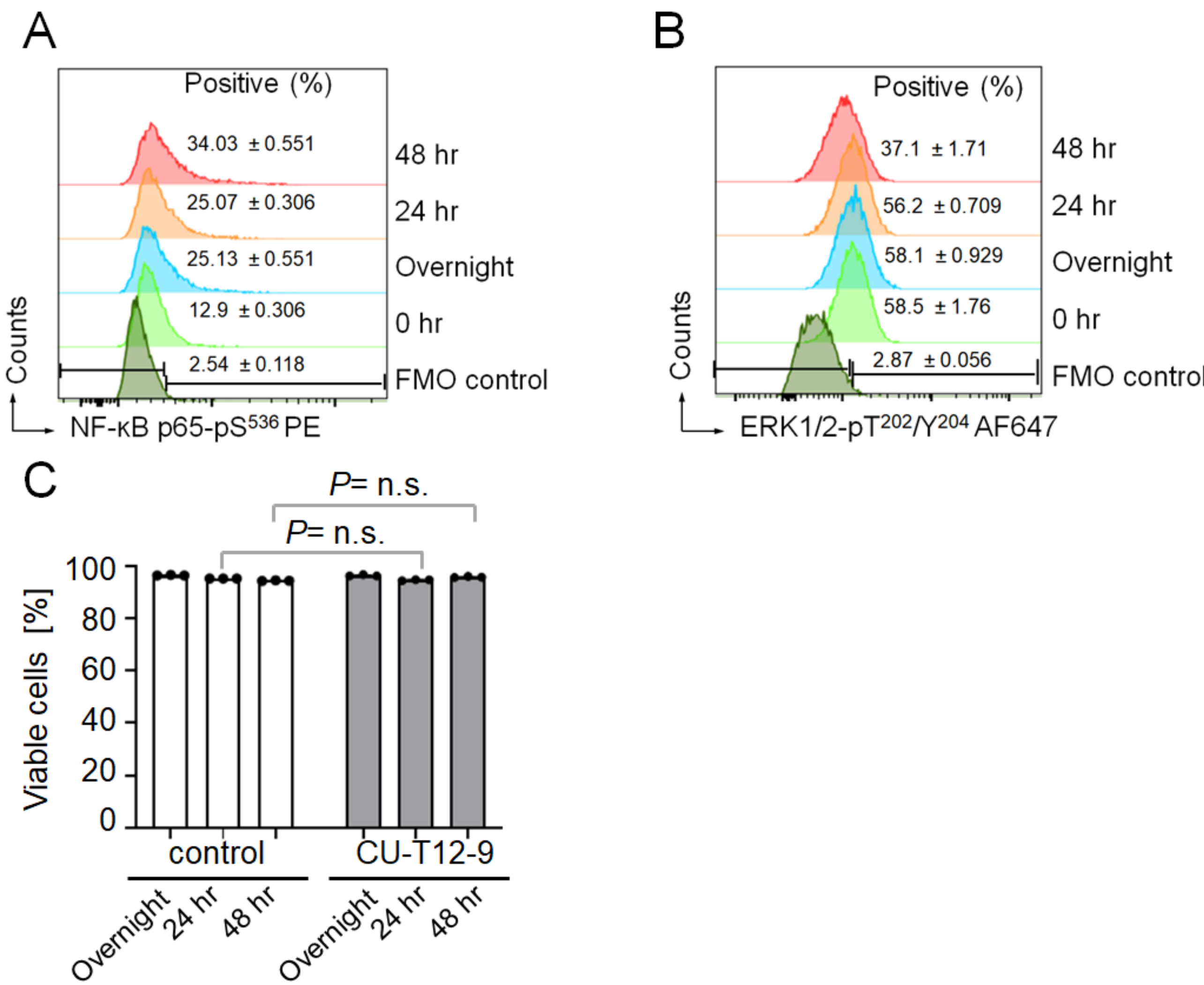

(A-B) Flow cytometry analyses of phospho-NF-κB p65 (Ser536; A) and phospho-ERK1/2 (Thr202/Tyr204; B) in 697 cells following treatment with CU-T12-9 (10 μM). Cells were stained with a PE-conjugated phospho-NF-κB p65 (Ser536) antibody or an AF647-conjugated phospho-ERK1/2 (Thr202/Tyr204) antibody. Quantified results are shown as mean ± s.d. from 3 independent experiments. FMO: Fluorescence minus one control (cells were stained with SYTOX® blue stain, a dead cell marker, but not PE-conjugated antibody against phospho-NF-κB or AF647-conjugated antibody phospho-ERK1/2). Statistical significance was determined by one-way ANOVA with Dunnett's multiple comparisons test. $P < 0.0001$ (A and B). (C) 697 cells were treated with either control or CU-T12-9 (10 μM). Cells were stained with SYTOX® Blue (a dead cell marker) at the indicated time points and their viability was assessed by flow cytometry. Cells stained negative for SYTOX® Blue were considered viable. Shown are results (mean ± s.d) from 3 independent biological replicates. Statistical significance was determined by Welch's t-test.

SUPPLEMENTARY TABLES

Table S1, *Overview of leukemia and lymphoma cell lines used in this study*

Cell line	Cancer type	Source	ID	Additional information
697	B-ALL, childhood	Creative Bioarray	CSC-C0217	NRAS^{G12D}; expression of fusion gene *TCF3-PBX* (*E2A-PBX*)
RS4;11	B-ALL, adult	ATCC	CRL-1873	*MLL-AF4* (*KMT2A-AFF1*)
Nalm-6	B-ALL, adult	ATCC	CRL-3273	NRAS^{A146T}; *ETV6-PDGFRB*
REH	B-ALL, childhood	ATCC	CRL-8286	*ETV6-RUNX1*
U2932	DLBCL	Creative Bioarray	CSC-C0624	
TMD8	DLBCL	Dr. Neetu Gupta, CCF		MYD88; p.Leu252Pro (c.755T>C) (L265P)
OCILY3	DLBCL	Dr. Neetu Gupta, CCF		MYD88; p.Leu252Pro (c.755T>C) (L265P)
SUDHL5	DLBCL	ATCC	CRL-2958	*CREBBP* deletion (loss of heterozygosity)

Notes: B-ALL: B-cell acute lymphoblastic leukemia; DLBCL: Diffuse large B-cell lymphoma; ATCC: American Type Culture Collection; CCF: Cleveland Clinic Foundation

Table S2, *Overview of genetic mouse models used in this study*

Mouse strain	Source	Purpose
C57BL/6J	The Jackson Laboratory	To study the consequences of canonical or non-canonical NF-κB activation
Nf1 23aIN/23aIN (Ref. 31)	Dr. Hua Lou, CWRU SOM	To model elevated RAS-ERK signaling

Notes: CWRU SOM: Case Western Reserve University School of Medicine

Table S3, *Mammalian gene expression vectors -regular plasmids and retrovirus constructs*

Construct	Expression of	Purpose
MSCV NRAS^{G12D}-Puro	NRAS^{G12D}; Puromycin resistance	Transformation; RAS-activation (retroviral transduction)
pCGDNSam-NRAS^{G12D}-IRES-Kusabira orange	NRAS^{G12D}; Kusabira orange	Transformation; RAS-activation (retroviral transduction)
pMIG EV	GFP	Empty vector control (retroviral transduction)
pMSCV IKBKB S177E/S181E EGFP	IKK2ca mutant, EGFP	Canonical NF-κB activation (retroviral transduction)
pMSCV MAP3K14 (NIK) EGFP	Wild-type NIK, EGFP	Noncanonical NF-κB activation (retroviral transduction)
pMIG IKBSR	Super-repressor form of IkBα, GFP	Modulation of NF-κB activity
pRP CMV EGFP	EGFP	Empty vector control (transfection)
pRP EGFP CMV IKBKB S177E/S181E	IKK2ca mutant, EGFP	Canonical NF-κB activation (transfection)
pRP CMV mCherry	mCherry	Empty vector control (transfection)
pRP mCherry CMV NRAS^{G12D}	NRAS^{G12D}; mCherry	Transformation; RAS-activation (transfection)
pLMN-ZsGreen-Neo	*Nfkb1*-trageting shRNA; ZsGreen; neomycin resistance cassette	*Nfkb1* knockdown (retroviral transduction)

Notes: NRAS^{G12D} constructs: gifts from Dr. Markus Müschen. The construct pLMN-ZsGreen-Neo was purchased from Transomic Technologies; other constructs were purchased from VectorBuilder.

Table S4, *Oligonucleotide sequences*

crRNA targeting human IGKC

crRNA	Sequence (5' to 3')
IGKC (crhIgK$_2$) (Ref. 48)	GTG GAT AAC GCC CTC CAA TC
IGKC (crhIgK$_3$) (Ref. 48)	CTG GGA GTT ACC CGA TTG GA
IGKC (crhIgK$_4$) (Ref. 48)	CCT CCA ATC GGG TAA CTC CC

shRNA targeting mouse Nfkb1

shRNA	Strand 5'	Strand 3'	Loop
Nfkb1	ATACCCTGAAATCAAAGACAAA	TTTGTCTTTGATTTCAGGGTAG	TAGTGAAGCCACAGATGTA
Nfkb1	CCTGGGCAAATGTTTCATTTGA	TCAAATGAAACATTTGCCCAGT	TAGTGAAGCCACAGATGTA
Nfkb1	ACAGACTGGCAGCTCTTCTCAA	TTGAGAAGAGCTGCCAGTCTGG	TAGTGAAGCCACAGATGTA

Table S5, *Antibodies used in this study*
Western blot antibodies

Antigen	ID	Manufacture
ERK-pT^{202}/Y^{204}	4370	Cell Signaling Technology
ERK	9102	Cell Signaling Technology
β-actin	3700	Cell Signaling Technology
NF-κB1 p105/p50	13586	Cell Signaling Technology
NF-κB p65-pS^{536}	3033	Cell Signaling Technology
NF-κB p65	8242	Cell Signaling Technology
BCL6	5650	Cell Signaling Technology
Zap-70 pY^{319} Syk p^{Y352}	2717	Cell Signaling Technology
Zap-70	3165	Cell Signaling Technology
Syk	13198	Cell Signaling Technology
FoxO1 pS^{256}	9461	Cell Signaling Technology
FoxO1	2880	Cell Signaling Technology
IκBα pS$^{32/36}$	9246	Cell Signaling Technology
IκBα	9242	Cell Signaling Technology

Flow cytometry antibodies for mouse cells

Antigen	Clone ID	Manufacturer
TruStain fcX Fc block		Biolegend
Ig light chain kappa	187.1	BD Biosciences
Ig light chain lambda	RML-42; R26-46	Biolegend, BD Biosciences
IgM	RMM-1	Biolegend
Cd127 (Il7r)	A7R34	Biolegend
Cd20	SA271G2	Biolegend
Cd40	3/23	Biolegend
Cd21	7E9	Biolegend
Cd23	B3B4	Biolegend

Flow cytometry antibody for human cells

Antigen	Clone ID	Manufacturer
Ig κ light chain	TB28-2	Biolegend; BD Biosciences
Ig λ light chain	MHL-38	Biolegend
Phospho-ERK1/2 (Thr202/Tyr204)	197G2	Cell Signaling Technology
Phospho-NF-κB p65 (Ser536)	93H1	Cell Signaling Technology
TruStain fcX Fc block		Biolegend
